# Prevalence of Household-level Food Insecurity and Its Determinants in an Urban Resettlement Colony in North India

**Published:** 2014-06

**Authors:** Palanivel Chinnakali, Ravi P. Upadhyay, Deepa Shokeen, Kavita Singh, Manpreet Kaur, Arvind K. Singh, Anil Goswami, Kapil Yadav, Chandrakant S. Pandav

**Affiliations:** ^1^Department of Preventive and Social Medicine, Jawaharlal Institute of Postgraduate Medical Education and Research (JIPMER), Puducherry, India; ^2^Department of Community Medicine, Vardhman Mahavir Medical College and Safdarjung Hospital, New Delhi, India; ^3^Institute of Home Economics, University of Delhi, New Delhi, India; ^4^International Food Policy Research Institute, New Delhi, India; ^5^Sitaram Bhartia Institute of Science and Research, New Delhi, India; ^6^Center for Community Medicine, All India Institute of Medical Sciences, New Delhi, India

**Keywords:** Determinants, Food insecurity, Prevalence, Urban slum, North India

## Abstract

An adequate food intake, in terms of quantity and quality, is a key to healthy life. Malnutrition is the most serious consequence of food insecurity and has a multitude of health and economic implications. India has the world's largest population living in slums, and these have largely been underserved areas. The State of Food Insecurity in the World (2012) estimates that India is home to more than 217 million undernourished people. Various studies have been conducted to assess food insecurity at the global level; however, the literature is limited as far as India is concerned. The present study was conducted with the objective of documenting the prevalence of food insecurity at the household level and the factors determining its existence in an urban slum population of northern India. This cross-sectional study was conducted in an urban resettlement colony of South Delhi, India. A pre-designed, pre-tested, semi-structured questionnaire was used for collecting socioeconomic details and information regarding dietary practices. Food insecurity was assessed using Household Food Insecurity Access Scale (HFIAS). Logistic regression analysis was performed to determine the factors associated with food insecurity. A total of 250 women were interviewed through house-to-house survey. Majority of the households were having a nuclear family (61.6%), with mean family-size being 5.5 (SD±2.5) and the mean monthly household income being INR 9,784 (SD±631). Nearly half (53.3%) of the mean monthly household income was spent on food. The study found that a total of 77.2% households were food-insecure, with 49.2% households being mildly food-insecure, 18.8% of the households being moderately food-insecure, and 9.2% of the households being severely food-insecure. Higher education of the women handling food (OR 0.37, 95% CI 0.15-0.92; p≤0.03) and number of earning members in the household (OR 0.68, 95% CI 0.48-0.98; p≤0.04) were associated with lesser chance/odds of being food-insecure. The study demonstrated a high prevalence of food insecurity in the marginalized section of the urban society. The Government of India needs to adopt urgent measures to combat this problem.

## INTRODUCTION

Food security exists when all people, at all times, have physical and economic access to sufficient, safe, and nutritious food that meets their dietary needs and food preferences for an active and healthy life (1). Food security can be examined at various levels, i.e. global, national, regional, household, and individual. Food security at the national or regional level does not necessarily indicate food security among communities, households, and individuals. Achievement of household food security does not necessarily account for the security at individual levels because of factors, such as gender discrimination (2-4).

Malnutrition is the most serious consequence of food insecurity. Adult malnutrition results in lower productivity on farms and in the labour market (5). In women, it also results in foetal malnutrition and low birthweights of babies (6). Childhood nutritional deficiencies are responsible, in part, for poor school enrollment, absenteeism, early dropout, and poor classroom performance, with consequent losses in productivity during adulthood (7-9). Not only does food insecurity in itself has deleterious effects on households and individuals but efforts at achieving food security may also pose a heavy economic toll if households must spend most of their income on obtaining food. On a household level, presence of food insecurity probably suggests a high degree of vulnerability to a broad spectrum of consequences, including psychosocial dysfunction in children, socio-familial problems, and overall poor health status.

The State of Food Insecurity in the World (2012) estimates that India is home to more than 217 million undernourished people (10). The Global Hunger Index (GHI) for India in the year 2010 was 24.1, which placed it in the “alarming category” (ranked 67, well below neighbouring countries, like China and Pakistan) (11). According to the National Family Health Survey (NFHS) Report-3, 33% of the women had their body mass index (BMI) below normal, and 45.9% of the children below the age of 3 years were underweight or malnourished (12).

India has among the world's largest urban population with below poverty line incomes and the world's largest population living in slums (13). The 2001 Census puts the slum population at 42.6 million which constituted 15% of the country's total urban population (14). In Delhi, the 2001 Census estimated an urban slum population of 1.85 million, which was 18.7% of Delhi's urban population (13). Urban slums have an underserved population and face a variety of problems, such as lack of sanitation and hygiene, food and water supply, and other basic amenities (15,16).

Various studies have been conducted to assess food insecurity at the global level; however, the literature is limited as far as India is concerned (17-23). Lack of sufficient studies on the burden of the problem poses a hurdle in formulating strategies to combating this epochal issue. Taking all this into consideration, the present study was conducted with the objective of documenting the prevalence of food insecurity at the household level in an urban resettlement colony in North India and the factors determining its existence.

## MATERIALS AND METHODS

### Study setting

The study was carried out in Dakshinpuri Extension, Dr. Ambedkar Nagar, which is a resettlement colony located in South Delhi. The study area has six blocks with 2,868 houses and a total population of about 20,000.

### Study participants

Females aged 18-50 years, who were largely responsible for handling the food preparation and food distribution within the household, were selected for the study. “Largely responsible” meant that the female handled the food preparation and food distribution for more than 2 complete meals a day and at least 5 days a week within the household.

### Sample-size and sampling design

Considering the prevalence of food insecurity in urban India as 44%, a relative precision of 20%, design effect of 2, and a non-response rate of 10%, the sample-size came to be 278 (24).

Out of the six blocks catering to a population of about 20,000, two blocks, i.e. Block 1 and Block 2, were selected randomly through lottery system. A list of eligible households having females (aged 18-50 years) was made from both the blocks. A total of 1,549 eligible households were listed (861 and 688 households from Block 1 and Block 2 respectively).

Using systematic random sampling with sampling interval of five, every 5th household from the prepared list of eligible households was selected. A total of 309 households (172 and 137 from Block 1 and Block 2 respectively) were finalized. If there were more than one eligible woman in a selected household, one woman was selected randomly from among them through lottery.

### Study instruments used

A predesigned, pretested, semi-structured questionnaire was used for collecting socioeconomic details and information regarding dietary practices. Socioeconomic details mainly comprised data on subject's age, education, religion, occupation, working status and nature of work, family type and size, family's total monthly income, and the approximate monthly expenditure on food. The total monthly family income and monthly expenditure on food were self-reported. A food frequency questionnaire was used for obtaining information about usual food consumption pattern in the household. The amount of raw food consumed by the members of the household was recorded as reported by the study participants. The participants were asked to report the amount in terms of the premeasured utensils that the data collectors were carrying (katoris, utensils, etc.), and then this amount was converted in grammes. The Household Dietary Diversity Scale (HDDS) was used for assessing the dietary diversity within the household. The participant was then interviewed according to the schedule. The height was measured in centimetres up to the precision of 0.1 cm, using adult portable stadiometer. Seca digital weighing scales were used in measuring weight of the individuals.

The Household Food Insecurity Access Scale (HFIAS), which is an adaptation of the approach used in estimating the prevalence of food insecurity in the United States (USA), was used in the present study (25). The method is based on the idea that the experience of food insecurity (access) causes predictable reactions and responses that can be captured and quantified through a survey and summarized in a scale. The HFIA prevalence indicator categorizes households into four levels of household food insecurity (access): food-secure and mildly, moderately and severely food-insecure. Households were categorized as increasingly food-insecure as these responded affirmatively to more severe conditions and/or had experienced those conditions more frequently.

The questions contained in the Household Food Insecurity Access Scale (HFIAS) were asked with a recall period of four weeks (30 days). The respondent was first asked an occurrence question, i.e. whether the condition in the question happened at all in the past four weeks (with the provision of ‘yes’ or ‘no’ response). If the respondent answered ‘yes’ to an occurrence question, a frequency-of-occurrence question was asked to determine whether the condition happened rarely (once or twice), sometimes (three to 10 times), or often (more than 10 times) in the past four weeks.

The operational definitions used in the current study were as follows:

*Food-secure:* A household was labelled ‘food-secure’ when the members ‘rarely’, in the past four weeks, worried about not having enough food and had replied ‘no’ to question number 2 to 9 (Table 1)
*Mildly food-insecure:* The members of the household worried about not having enough food sometimes or often, and/or were unable to eat preferred foods, and/or ate a more monotonous diet than desired, and/or ate some foods considered undesirable but only rarely (25).
*Moderately food-insecure:* The household members sacrificed quality more frequently by eating a monotonous diet or undesirable foods sometimes or often, and/or had started to cut back on quantity by reducing the size of meals or number of meals, rarely or sometimes (25).
*Severely food-insecure:* The individuals in the household had to cut back on meal-size or number of meals often, and/or experienced any of the three most severe conditions (running out of food, going to bed hungry, or going a whole day and night without eating) (25).

### Data collection

A team of three trained postgraduate students in the discipline of nutrition collected data for the study under the supervision of the Community Medicine Faculty and resident doctors. The questionnaire was pretested, responses discussed amongst authors, and modified to ensure standardization. House-to-house survey was done. Participants were provided with the information sheet in local language (Hindi), and they were explained about the study, its objective, procedure, and their rights. If the participant agreed to participate in the study after going through the information sheet, a written consent in Hindi was taken. If the subject was unavailable or pre-occupied during the first visit, the interviewers set a date and time for the next two visits. The participants refusing to participate were categorized as ‘non-respondents’.

The study was conducted in compliance with ‘Ethical Principles for Medical Research Involving Human Subjects’ of the Helsinki Declaration. Departmental ethical clearance from the committee comprising all the faculty members of Centre for Community Medicine was obtained before starting the study. Confidentiality of each participant was ensured, and any possible ethical concerns were discussed prior to starting the survey. The researchers shared their identity and contact details with the participants and encouraged them to contact in case of any issues concerning their participation in the study.

**Table 1.** Prevalence of food insecurity, based on Household Food Insecurity Access Scale, among households in an urban resettlement colony in South Delhi, India

Question	Rarely	Sometimes	Often
	n (%)	n (%)	n (%)
1. In the past four weeks, did you worry that your household would not have enough food?	57 (22.8)*	5 (2)†	0 (0)†
2. In the past four weeks, were you or any household members not able to eat the kinds of foods you/they preferred because of a lack of resources?	24 (9.6)†	4 (1.6)†	0(0)†
3. In the past four weeks, did you or any household members have to eat a limited variety of foods due to a lack of resources?	7 (2.8)†	1 (0.4)‡	0 (0)‡
4. In the past four weeks, did you or any household members have to eat some foods that you/they really did not want to eat because of a lack of resources to obtain other types of food?	83 (33.2)†	1 (0.4)‡	0 (0)‡
5. In the past four weeks, did you or any household members have to eat a smaller meal than you/they felt you/they needed because there was not enough food?	23 (8.9)‡	1 (0.4)‡	0 (0)¶
6. In the past four weeks, did you or any household members have to eat fewer meals in a day because there was not enough food?	19 (7.6)‡	2 (0.8)‡	0 (0)¶
7. In the past four weeks, was there ever no food of any kind to eat in your household because of lack of resources to get food?	16 (6.4)¶	5 (2)¶	0 (0)¶
8. In the past four weeks, did you or any household members go to sleep at night hungry because there was not enough food?	1 (0.4)¶	1 (0.4)¶	0 (0)¶
9. In the past four weeks, did you or any household members go a whole day and night without eating anything because there was not enough food?	0¶	0¶	0 (0)¶

*Food-secure;

†Mildly food-insecure;

‡Moderately food-insecure;

¶Severely food-insecure

### Data analysis

Data were entered in Microsoft Excel and subsequently transferred to SPSS (version 17) (Chicago, IL, USA) for statistical analysis. Wherever applicable, proportion and mean (SD) were calculated. One-way ANOVA was used in detecting any statistically significant difference in terms of consumption of various food-groups between the HFIAS categories. Afterwards, the four HFIAS categories were grouped into two (food-secure/mildly food-insecure and moderately/severely food-insecure); independent Student's *t*-test was used in looking for any significant difference in terms of consumption of different food-groups. Logistic regression analysis was performed to determine the factors associated with food insecurity. The regression analysis was performed considering outcome in dichotomous form, i.e. food-secure and food-insecure. The food-insecure group included mild, moderate and severe food insecurity. Initially, each independent variable was regressed against each dependent variable. Those variables with a minimum p value of 0.25 were considered for multiple logistic regression analyses (26-28). All the predictor variables that were significant in bivariate analysis were entered in the model and regressed using stepwise backward elimination. A p value of <0.05 was finally considered to be statistically significant in the multivariate model. All the analyses were done by the faculty and postgraduate resident doctor(s) of the Centre for Community Medicine, who were trained in epidemiology and biostatistics (PC, RPU, and KY).

The dietary data on the households were expressed in consumption units (CU) based on different coefficients for practical nutrition work in India by Gopalan *et al*. (29). Further, intake of different food substances (such as cereals, pulses, fruits, vegetables, milk, etc.) per consumption unit was computed using the following formula:


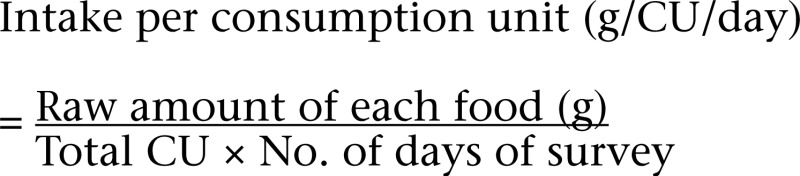


## RESULTS

A total of 309 households were eligible for the study, and 309 females were contacted but, in only 250 of them, the females had consented for participation in the study. The reason for non-response was that the eligible females were pre-occupied in household work at the time of interview. Thus, the response rate in the present study was 81%.

### Sociodemographic profiles

The sociodemographic profiles of the study participants are shown in Table 2. The mean age of the respondents was 33±8 years. Most of the households followed Hinduism (94.8%). Majority of the households were having a nuclear family setting (61.6%). The mean family-size was 5.5±2.5. Around 45% of the households had 5-7 members in their family. The mean number of earning members in the households included in the study was 1.59±0.9. Nearly 59% of the households had one earning member while those with two earning members were around 28%. The mean monthly household income reported by the respondents was INR 9784±631 (US$ 191.5±12.4). Around one-fourth (27.2%) of the households had a monthly income of less than Rs. 5,000. The mean BMI was 23.7±4.1 kg/m^2^ with 7.6% study participants being thin (BMI <18.5 kg/m^2^) and 33.2% being overweight (BMI >25 kg/m^2^).

### Food consumption pattern

Out of 250 households included in the study, more than 90% were vegetarians (n=227). In majority of the households, i.e. 97.6%, the members had ≤2 meals a day. Majority of the households had cereals as their staple food (99.2%). Further, 73.2% of the households consumed vegetables, and 70.8% consumed pulses daily. In around four-fifths of the households (77.6%), home-cooked food was preferred while there were 22% households in which both home-cooked and food from outside was preferred.

**Table 2.** Key sociodemographic profile of the study respondents (N=250)

Variable	Number	Percentage
Age of study respondents (in completed years)		
18-28	88	35.2
29-39	99	39.6
40-50	63	25.2
Religion		
Hindu	237	94.8
Muslim	08	3.2
Sikh	04	1.6
Christian	01	0.4
Type of family		
Nuclear	154	61.6
Joint	96	38.4
Family-size		
≤4	102	40.8
5-7	111	44.4
≥8	37	14.8
House		
Own	207	82.8
Rented	43	17.2
Educational status of study respondents		
Non-literate	38	15.2
Primary	31	12.4
Middle	48	19.2
Secondary	77	30.8
Senior secondary	33	13.2
Graduate	23	9.2
Monthly family income (in Rupees)		
<5,000	68	27.2
5,001-10,000	120	48
10,001-20,000	51	20.4
20,001-25,000	7	2.8
>25,000	4	1.6

### Prevalence of food insecurity

In the present study, it was found that 77.2% of the households were food-insecure (Table 3). Around half (49.2%) of the households were mildly food-insecure. Nearly one-fifth (18.8%) of the households faced moderate food insecurity (Table 3). Overall, 9.2% of the households were found to be severely food-insecure.

Table 1 further shows that, in the past four weeks, 9.6% of the households, ‘rarely’ and 1.6% of the households, ‘sometimes’ were not able to eat the kinds of food they preferred. Also, 2.8% of the households ‘rarely’ had to eat a limited variety of foods; 33.2% of the households ‘rarely’ and 0.4% of the households ‘sometimes’ had to eat some foods that they really did not want to eat. Around 9% of the households ‘rarely’ had to eat a smaller meal than they felt they needed because there was not enough food.

### Utilization of public food distribution system

Around two-thirds (62.8%) of the respondents had a ration card. Most of them (53.2%) were not regularly availing service of the public food distribution system (PDS) in spite of owning a ration card. The main reason for this was the insufficient quantity given and poor quality of grains provided to them as ration. Instead of availing the PDS services, most of the households preferred buying ration from the retail shops and private ration shops. Majority (75.3%) of those availing PDS service were not getting the adequate ration for their families.

**Figure.**
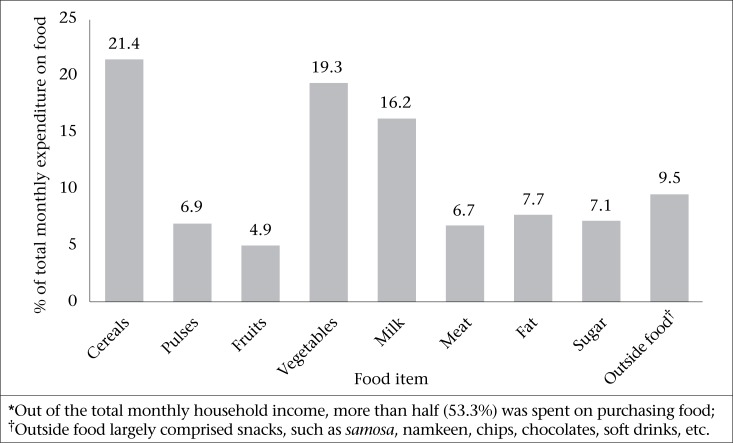
Graphical representation of the monthly household expenditure on different food-groups as a percentage of the total monthly expenditure on purchasing food*

**Table 3.** Summary statistics showing the prevalence of different levels of food insecurity among households in an urban resettlement colony in South Delhi, India (N=250)

Household food insecurity level	No. of households	Percentage
Food-secure	57	22.8
Mildly food-insecure	123	49.2
Moderately food-insecure	47	18.8
Severely food-insecure	23	9.2

**Table 4.** Food intake per consumption unit according to Household Food Insecurity Access Scale (HFIAS) category

HFIAS category*	Food intake per consumption unit (g/CU/day)†							
	Cereals	Pulses	Fruits	Vegetables	Milk	Meat	Oil‡	Sugar
Food-secure	297.2±141.1	40.3±39.7	64.7±49.7	198.2±122.3	281.8±136.2	42.3±78.5	45.3±27.4	47.3±31.3
Mildly food-insecure	282.5±117.5	41.0±37.4	66.4±64.3	224.3±145.4	284.4±149.2	24.2±61.7	37.0±21.5	40.3±35.3
Moderately food-insecure	281.5±124.1	40.4±44.5	59.6±66.9	183.6±126.8	238.2±119.7	15.4±18.9	34.2±16.8	40.2±49.5
Severely food-insecure	313.4±196.7	39.3±29.4	51.3±65.4	185.4±127.5	237.1±146.5	14.8±20.3	33.0±20.9	43.3±26.8

*One-way ANOVA used in detecting any statistically significant difference in terms of consumption of various food-groups between the HFIAS categories;

†Data presented as mean±SD;

‡Statistically significant differences observed between the four groups under HFIAS categories (p=0.03)

### Purchasing power and monthly expenditure on food

In the present study, the mean total monthly income of the household was Rs. 9784±631, out of which a major proportion was spent on purchasing food (53.3%). The mean monthly household income for food-secure and food-insecure households was Rs. 14210.5±1744.6 and Rs. 8476.7±899.1 respectively. The mean amount spent monthly on food by food-secure households was Rs. 7245.6±581.8 which was higher than that spent by food-insecure households, i.e. Rs. 4614.2±162.1 (p=0.001). Although not statistically significant, food-insecure households spent a comparatively more percentage of the monthly family income on food (55.34%) than the food-secure households (47.63%) (p=0.71). The proportion of monthly expenditure on different food-groups, out of the total monthly expenditure on food per household, has been shown in the Figure. It is evident that the monthly expenditure on food-groups, like cereals (21.4%), vegetables (19.3%), and milk (16.2%), was more compared to other food-groups, like pulses (6.9%), fruits (4.9%), and meat (6.7%). Also, expenditure on other food items, like sugar, fat, and convenient foods (such as noodles, chips, chocolates) was relatively high.

### Food intake per consumption unit according to HFIAS category

It is evident from Table 4 that, except for oils, the daily intake (in grammes) per consumption unit for all other food items was not statistically significant among the four HFIAS categories. On combining the food-secure/mildly food-insecure into one category and moderately/severely food-insecure in another category, it was found that, except for vegetables (216.0±138.8 vs 184.2±126.1; p=0.04), milk (283.6±144.8 vs 237.8±128.1; p=0.007), and oil (39.6±23.7 vs 33.8±18.1; p=0.01), the daily intake (in grammes) per consumption unit for cereals, pulses, fruits, meat, and sugar was not statistically significant among the two groups.

### Determinants of food insecurity

The variables significantly associated with food insecurity have been shown in Table 5. The households with the education of the respondent, i.e. females responsible for food-handling having primary or middle school education (OR 0.30, 95% CI 0.10-0.90; p≤0.03) and secondary or senior secondary school education (OR 0.37, 95% CI 0.15-0.92; p≤0.03) had less chances of being food-insecure compared to those households where the respondent was non-literate. Also, compared to households with per-capita monthly income between Rs. 1,000 and 2,000, those with per-capita monthly income of less than or equal to Rs. 1,000 had higher chances of being food-insecure (OR 4.77, 95% CI 1.66-13.65; p≤0.004). With a unit increase in the number of working members in a household, the chances of being food-insecure decreased by 0.32 (p=0.04).

**Table 5.** Determinants of food insecurity in an urban resettlement colony in South Delhi, India

Variable*	Adjusted OR	95% CI	p value
Education of respondent†			
Primary and middle school	0.30	0.10-0.90	0.03
Secondary and senior secondary school	0.37	0.15-0.92	0.03
Graduate and above	0.18	0.05-0.69	0.01
Non-literate	1		
Per-capita monthly income (in Rupees)			
≤1,000	4.77	1.66-13.65	0.00
2001-3,000	0.80	0.32-2.00	0.63
>3,000	1.01	0.36-2.82	0.97
1,001-2,000	1		
Number of working members in the household‡	0.68	0.48-0.98	0.04

*Only the variables that were significantly associated with food insecurity have been presented in the table. Other variables included in the analysis were education of the head of the household, type of family (nuclear/joint), family-size, type of house (owned/rented), religion, and utilization of the public food distribution system (PDS);

†The females participating in the study, who were largely responsible for the food preparation and distribution within the household;

‡Considered a continuous predictor variable in logistic regression analysis

## DISCUSSION

Food is a basic necessity of life and essential for sustenance. An adequate food intake, in terms of quantity and quality, is a key for healthy life. The current study looked at household-level food insecurity in an urban resettlement colony in North India, using a valid and reliable tool, i.e. Household Food Insecurity Access Scale (HFIAS). The study found that a total of 77.2% households were food-insecure, be it mildly (49.2%), moderately (18.8%), or severely (9.2%). The level of food insecurity reported in the current study is much higher than in previous studies done in other parts of the country (22,23,30,31). While the methodology of assessment of food security and the instruments used were different in previous studies, possible reasons for the difference could be the average family-size of 5.5, mostly a single working member in the household and a low monthly income in the current study.

Similar to that documented by Ray *et al*. (1997 and 2000), the present study showed that utilization of public food distribution system (PDS) was low; also, the quantity of ration provided did not meet the specified amount (22,32). This could have played a role in coercing the households to depend on other unauthorized sources for meeting their requirements, which might have further led to food insecurity largely due to economic constraints. It would not be out-of-context to restate the importance of measures to strengthen PDS and make it available to those who fall under its scope.

Our results show that, among households surveyed, low monthly per-capita income was one of the significant independent predictors of food insecurity. Also, with increase in the number of working members, the odds of a household being food-insecure decreased. These findings offer evidence for implementing sustainable employment-generation initiatives in order to ensure economic stability and, which helps in making the households food-secure. Employment-generation schemes, such as Mahatma Gandhi National Rural Employment Guarantee Act (MGNREGA) for rural population need to be introduced and upscaled in urban population also so as to make a positive impact on the purchasing power of the lower socioeconomic segment of the population (16).

The study found that around half of the monthly income was spent on purchasing food and, even then, there was a high prevalence of food insecurity. It might be due to the large family-size where the amount spent on food would have been insufficient to meet the food requirements of all the family members. Although the main part of the income was spent on the food procurement, ironically, the quality of the food consumed was compromised as respondents reportedly consumed more food of cheap carbohydrate sources and relatively little of pulses and fruits. This finding reflects the gap in knowledge of the community members in the study area regarding healthful food sources and to fill the same, undertaking awareness-creating campaigns becomes a priority.

### Strengths and limitations

The strength of the study lies in being a community-based study with a considerably high participation rate. Also, this is probably amongst the few studies done in an urban resettlement colony in North India to document the status of food security/insecurity at the household level. The study had its limitations too. In the present study, food security was a direct measure of the household's ability to afford food, and the scale used did not consider other aspects which probably have a bearing on food security, such as gender discrimination in food allocation, quality of the food consumed, food fads, and preferences. Therefore, these aspects would need to be explored more, along with the dynamics of intra-familial food distribution. The sample-size was calculated considering a non-response rate of 10% but, during the data collection, the non-response rate was more than that. This might reduce the precision of the findings. Also, the primary reason for non-response was that the respondents were pre-occupied with household work at the time of interview. Pre-occupied women could be more food-secure, and this might have led to an overestimated food insecurity rate. Information on monthly family income and expenditure on food was self-reported and could be subject to bias.

### Conclusions

The current study found that there was a high prevalence of food insecurity in an urban resettlement colony of Delhi. More such studies are required to generate enough evidence to influence policy so that measures are taken against this food insecurity problem in the already-marginalized section of the urban society. The Government needs to supplement the provision of food security (through a universal or targeted approach) with a mix of short- and long-term policies. The short-term policies could include improving the poor environmental conditions, ensuring ample employment opportunities and strengthening the public food distribution system. Long-term intervention should focus more on intersectoral coordination, involvement of non-governmental organizations, and ensuring women's empowerment.
